# Supplementary material for: The numerical simulation of droplet impact on surfaces is conducted using the SPH-DEM method
Source: PLoS One. 2025 Jun 2;20(6):e0323418. doi: 10.1371/journal.pone.0323418 (PMC12129337; doi:10.1371/journal.pone.0323418)
Supplement: Attachment — Submitted filename: Comments on PONE-D-24-51781.pdf [file pone.0323418.s001.pdf]

In my opinion, this study proposed a coupled SPH-mesh method to simulate the interaction between cloth and water. The method is novel for me, and the application is interesting. However, the paper is not written well. So, it is difficult for me to understand the proposed method. I suggest a major revision before publication. The suggestions are following as:

1. According to lines 74-78 of page 3, I guess the authors used three types of particles to simulate the cloth, the fluid, and the porous materials. I suggest the authors use fixed terminology to describe these three types of particles separately, and use a unified terminology in the entire text. For example, the cloth is denoted by cloth node, the water is denoted by fluid particle, and the porous material is denoted by porous particles. The authors should avoid to use SPH particle which is very vague.
2. According to figures 1 and 2, I guess the authors used mesh method to simulate the cloth, not SPH method. If the mesh method is used, the authors should introduce the used mesh method. And, the authors should provide some comments on the recent coupled SPH-mesh methods, such as the papers by Huang et al. (https://doi.org/10.1016/j.enganabound.2022.02.011) and He et al. (https://doi.org/10.1016/j.coastaleng.2024.104569).
3. The cloth is very thin, and belongs to ultra-thin flexible elastic material. How did the authors calculate the deforming of the cloth? How did the authors deal with the thin boundary condition? For the simulation of the ultra-thin flexible elastic material, the recent paper by Bao et al. (https://doi.org/10.1016/j.cma.2024.117255) proposed an effective SPH model, which may be referenced by the authors.
4. I suggest the authors use fixed symbols to denote the fixed type particles. For example, the symbols i and j are used to denote the water particles, the symbols n and m are used to denote the cloth nodes, the symbols k and l are used to denote the porous particles. These symbols should be unified in the whole text.
5. Please explain each equation and provide an explanation for each variable in the equation. For example, how to solve the variables w of equation (1) and $m_{n,f}$ of equation (2)? How to derive the equation (6) from (4)? Additionally, the Eq. (4) used in this study is similar to the seepage model in soil mechanics by He et al. (https://doi.org/10.1016/j.enganabound.2024.105755). In He et al.'s work, the interaction between water and soil is calculated by the Darcy's law, however the water pressure in the soil is interpolated from the information of the water particles. In this study, is the eq. (5) similar to the interpolation?
6. The authors are required to carefully check and polish the writing of the paper because there are many careless misprints. For example, there are some strange symbols in table 2.
7. The authors are suggested to provide the initial particle distribution, and provide the schematic diagram of computational model.
8. The authors are suggested to enhance the introduction for SPH in the field of fluid and solid interaction (FSI). There are many SPH papers which are related with FSI. For example, Wang et al. (https://doi.org/10.1063/5.0236386) used SPH method to simulate the swimming fish school.
