# Supplementary material for: The numerical simulation of droplet impact on surfaces is conducted using the SPH-DEM method
Source: PLoS One. 2025 Jun 2;20(6):e0323418. doi: 10.1371/journal.pone.0323418 (PMC12129337; doi:10.1371/journal.pone.0323418)
Supplement: Attachment — Submitted filename: Response to Reviewers.docx [file pone.0323418.s003.docx]

Dear Editors and Reviewers:

The co-authors and I would like to thank you for the precious time and effort spent in reviewing the manuscript entitled “**The numerical simulation of droplet impact on surfaces is conducted using the SPH-DEM method**” (Manuscript No. PONE-D-24-50755).

Those comments are all very valuable and helpful for revising and improving this manuscript, as well as the important guiding significance to our researches. We have studied every comment carefully and have made correction which we hope meet with approval. Revised portion are marked in yellow highlight in the manuscript. The main corrections in the manuscript and the responds to the reviewers’ comments are as flowing：

**Reviewer #1:**

1. The literature review is not comprehensive. It would benefit the discussion of recent reports on the simulation of droplets impact, e.g.

10.1007/s10409-024-24165-x

10.1016/j.oceaneng.2024.118730

10.1016/j.ijadhadh.2024.103734

**Answer**：Thank you for pointing out the deficiencies in the literature review and providing crucial references. We have carefully studied these three recent publications and incorporated their core contributions into the discussion section of the revised manuscript to enhance both the comprehensiveness and cutting-edge nature of this study. Below are the specific additions and modifications made:

In recent years, droplet impact simulation methods have achieved remarkable progress in multi-physics field coupling and complex interface handling. For instance, He et al. [25] revealed the aggregation effect of liquid metal droplets on elastic substrates through SPH method, providing novel insights into energy dissipation mechanisms at soft material interfaces; Dong et al. [26] developed a dual-core SPH model that effectively suppresses tensile instability, laying methodological foundations for contact time regulation of macrostructurally structured surfaces; Meanwhile, Huo et al. [27] comparatively demonstrated the robustness of Lagrangian framework-based fluid-solid coupling simulations by contrasting FEM-SPH and SPH-SPH approaches in elastic beam vibration analysis.

[25] He, Z., Xiao, R. & Qu, S. A study on the impact of liquid metal droplets onto metal and elastomer substrates. Acta Mech. Sin. 41, 224165 (2025). https://doi.org/10.1007/s10409-024-24165-x

[26] Huo, Y., et al., Modeling and simulation of droplet impact on an elastic beam based on FEM-SPH and SPH-SPH FSI methods. Ocean Engineering, 2024. 310: p. 118730.

https://doi.org/10.1016/j.oceaneng.2024.118730

[27] Dong, X., L. Feng and Q. Zhang, Droplet asymmetry bouncing on structured surfaces: A simulation based on SPH method. International Journal of Adhesion and Adhesives, 2024. 132: p. 103734. https://doi.org/10.1016/j.ijadhadh.2024.103734

2. Amend r_ij = |r_ij|

**Answer**：The relevant formulas have been modified in the revised manuscript.

***r****_ij_*=∣*r_ij_*∣

3. eta = 0.01 h^2 is not added in the equation.

**Answer**：Thank you for your meticulous review. Equation (6) in the original manuscript did contain a typographical error. We have conducted a thorough re-examination of the equations and implemented necessary corrections.

$\frac{d\mathbf{v}_{i}}{dt}=\text{ }\mathbf{g}_{i}-\sum_{j} m_{j}\left(\frac{P_{i}}{\rho_{i}^{2}}+\frac{P_{j}}{\rho_{j}^{2}}\right)\nabla_{i}W_{ij}\text{ }+\sum_{j} \frac{2m_{j}\mu\mathbf{r}_{ij}\cdot\nabla_{i}W_{ij}}{\rho_{i}\rho_{j}\left(r_{ij}^{2}+\eta\right)}\mathbf{v}_{ij}$ （6）

4. In the coupling procedure, clarify the role of the adsorption force induced by capillary action, as it seems relevant in light of the claimed application concerning the droplet impact on surfaces.

**Answer：**Thank you for your attention to the role of adsorption forces in the coupled model. The capillary-induced adsorption force plays a crucial role in the dynamic process of droplet impact on particle surfaces, particularly during the stages of droplet spread, retraction, and particle engulfment. The following sections will elaborate on the physical significance of adsorption forces and their impact on droplet behavior from two aspects:

**(1) The mathematical model of adsorption force and its physical significance**

In the SPH-DEM coupling procedure, the adsorption force induced by capillary action plays a critical role in governing the interaction between the droplet and particle surfaces. This force arises from the capillary pressure within the interstitial voids between particles, which is influenced by the wettability of the particles and the local saturation of the fluid. The adsorption force is modeled to account for capillary effects caused by fluid infiltration into the gaps between particles. This force acts as an indirect attraction between the fluid and solid particles, driven by the wetting properties of the particles (quantified by the wetting coefficient ζ) and the pore pressure near the particle surfaces. The wetting coefficient is determined by the particle material property and the local saturation (Sa), which is calculated based on the density of fluid particles within the support domain (Eq. 21–22). The adsorption force is integrated into the SPH-DEM coupling equations (Eq. 20, 23–24) as part of the total interaction force between fluid and solid particles. It directly affects the momentum exchange between the droplet and particles during spreading and retraction phases. For instance, during droplet spreading, capillary forces enhance fluid-particle adhesion, while during retraction, they contribute to the "pull-back" effect driven by surface tension, influencing droplet rebound dynamics.

**(2) Impact on Droplet Behavior:**

**Spreading Phase**: The adsorption force facilitates fluid infiltration into particle gaps, increasing the contact area and energy dissipation. This aligns with the observed dependency of maximum spreading diameter on particle size (Fig. 13), where smaller particles (e.g. *d*=20*μm*) exhibit faster retraction due to stronger capillary interactions.

**Retraction Phase**: The interplay between capillary forces and surface tension governs the rebound rate. Higher adsorption forces (e.g., in smaller particles or higher impact velocities) accelerate retraction by intensifying momentum exchange between fluid and particles (Fig. 11–13).

**Particle encapsulation phenomenon:** Adsorption forces drive the encapsulation of particles by the surface of a droplet (Fig. 10-12), simulating the experimentally observed "drop-encased particle" nucleation process. This mechanism is crucial for achieving uniform particle distribution in coating processes.

5. “The critical time step in the DEM model should be associated with the propagation speed of Rayleigh Waves along the surface of solid particles” This position should be strengthen at least by give References.

**Answer**：Thank you for your suggestion. We have already supplemented the relevant references in the manuscript.

[39] Rayleigh, L., (1885) On Waves Propagated along the Plane Surface of an Elastic Solid. Proceedings of the London Mathematical Society, s1-17(1): p. 4-11. https://doi.org/10.1112/plms/s1-17.1.4

6. Fig. 3 While wettability is overall in a fairly good agreement, the impact process returns different shapes in Fig. 2, if not below 4.5 ms. Energy conversion should be further investigated, along which numerical values of ruling parameters.

**Answer：**We appreciate the reviewer’s insightful observation regarding the differences in droplet shapes during the impact process (Fig. 2) and the need for further investigation into energy conversion and parameter sensitivity. Below, we address these points in detail:

**（1） Discrepancies in Droplet Shapes Below 4.5 ms (Fig. 2):**

The observed differences in droplet morphology during the early impact phase (0–4.5 ms) can be attributed to the following factors:

**a. Dynamic Surface Tension Effects:**

While the SPH model incorporates a surface tension term (Eq.7–9), the current implementation assumes a constant surface tension coefficient (σ=0.04N/m). However, during high-speed droplet impact, dynamic surface tension effects may arise due to rapid deformation, which are not explicitly modeled. This simplification could lead to deviations in droplet spreading dynamics compared to experiments.

**b. Viscous Dissipation and Strain Rate Dependence:**

The viscous term in Eq. 6 employs a constant dynamic viscosity. At the high strain rates encountered during impact, non-Newtonian behavior or strain-rate-dependent viscosity might influence energy dissipation. Future work will explore strain-rate-dependent viscosity models to better match experimental observations.

**c. Boundary Condition Simplifications:**

The hydrophobic wall is modeled using a fixed wetting coefficient (*ζ*) derived from saturation *Sa* (Eq. 21). However, transient contact angle hysteresis and slip boundary effects during rapid spreading are not fully resolved. Incorporating dynamic contact angle models (e.g., Cox-Voinov theory) could improve accuracy during the initial impact phase.

**（2）Energy Conversion Mechanisms**

Thank you for your insightful comments on the importance of studying energy conversion mechanisms. The issues you raised regarding energy transformation and key parameter quantification during droplet impact processes are indeed critical for gaining a deeper understanding of droplet dynamic behavior. We fully agree with your viewpoint and have incorporated this topic as a core focus for our future research. Below is our detailed explanation and implementation plan:

The SPH-DEM coupled model has preliminarily validated the correlation between droplet spreading-retraction behavior and surface tension, particle wettability (Fig. 8-9). However, the multiphase coupling between droplets and particulate beds involves dynamic interconversion among kinetic energy, surface energy, viscous dissipation, and particle frictional energy. To address this, we are developing specialized energy tracking algorithms (e.g., particle-level energy tagging method), which is currently under active development and debugging. The current version has not yet implemented systematic quantification of energy conversion processes.

In response to your valuable suggestion, we plan to integrate energy conservation equations into the SPH-DEM framework in our next phase of work. This will enable real-time calculation of four critical energy components during droplet-particle interactions:

Droplet kinetic energy (*E_k_*), Surface energy (*E_s_*), Viscous dissipation energy (*E_v_*), Particle frictional energy (*E_f_*). The governing equations will be formulated as follows:

$$E_{k}=\frac{1}{2}\sum m_{i}v_{i}^{2}, E_{s}=\sigma\cdot A, E_{v}=\int\mu(\nabla\mathbf{v})^{2}dt, E_{f}=\sum F_{ij}\cdot\delta_{ij}$$

where, *A* represents the drop surface area, *F_ij_* denotes the particle contact force, and *δ*_ij_ corresponds to the relative displacement. Subsequently, we will quantify the effects of the Weber number (*We*), particle diameter (*dp*), and wetting coefficient (*ζ*) on the energy allocation ratio, combined with theoretical models from literature for validation.

7. Fig. 4. Particle distribution along the deformed free surfaces seems to show excessive irregularity. Bouncing effects should be the cause. A further investigation of this behaviour would be needed.

**Answer**: We sincerely appreciate the reviewer’s observation regarding the irregular particle distribution along the deformed free surfaces in Fig. 4 and their suggestion to investigate potential bouncing effects. Below, we provide a detailed explanation and outline steps for further analysis:

The DEM model employs the Hertz-Mindlin contact theory (Eq. 16–19) with a damping coefficient derived from the restitution coefficient *e*=0.92 (Section 2.2). While this setup effectively captures energy dissipation during collisions, it may oversimplify microscale particle interactions in dense granular flows. Specifically, the linear spring-dashpot model assumes idealized elastic-plastic collisions, which might not fully resolve multibody collisions or particle rearrangement dynamics during rapid collapse.

We attempted to reduce the coefficient of restitution of the particles (*e*=0.8), and the simulation results showed that smoother particle distributions (attached supplementary figure). This suggests that optimizing damping parameters can mitigate irregularity.

（b）

The following research, we are going to address the bouncing effect through parameter optimization and the implementation of advanced contact mechanics, with the aim of enhancing the physical fidelity in particle dynamics simulations. We sincerely appreciate the reviewers' valuable feedback, which will significantly strengthen the robustness of our model.

8. Fig. 10. Droplet diffusion process. Molecular diffusion? Any validation?

**Answer：**Thank you for your question. Here we confirm that there are inaccuracies in the formulation of the ‘**droplet** **diffusion process**’ in Fig. 10. The term ‘diffusion’ in our manuscript refers to the macroscopic spreading of the droplet driven by inertia and surface tension, rather than molecular-scale diffusion processes. The latter is negligible in the studied millimeter-scale droplet impact dynamics due to the dominance of advection and capillary forces.

We made revisions to the relevant statements in the revised manuscript.

Thanks to you for your comments and suggestions again.

**Reviewer #2:**

1. In Eq. (6), what does the variable ν0 mean?,

**Answer:** Thank you for your meticulous review. Equation (6) in the original manuscript did contain a typographical error. We have conducted a thorough re-examination of the equations and implemented necessary corrections.

$$\frac{d\mathbf{v}_{i}}{dt}=\text{ }\mathbf{g}_{i}-\sum_{j} m_{j}\left(\frac{P_{i}}{\rho_{i}^{2}}+\frac{P_{j}}{\rho_{j}^{2}}\right)\nabla_{i}W_{ij}\text{ }+\sum_{j} \frac{2m_{j}\mu\mathbf{r}_{ij}\cdot\nabla_{i}W_{ij}}{\rho_{i}\rho_{j}\left(r_{ij}^{2}+\eta\right)}\mathbf{v}_{ij}$$

where **v***_ij_*= **v***_i_*-**v***_j_*, **r***_ij_* =**r***_i_*-**r***_j_*, *r_ij_* = |**r***_ij_*|. *h* is the smoothing length. The third term on the right side of the Eq. (6) is the viscous term. *μ* is the dynamic viscosity of the fluid. **g** denotes the body force acting on the fluid, such as gravitational force. The term *η* = 0.01h^2^ was added to prevent the singularity of the viscous term when two particles approach each other infinitely.

2. For the free surface flow, it usually uses the so-called artificial viscous scheme to discretize the viscous term of fluid instead of the physical viscous scheme because the artificial viscous scheme can satisfy the free surface boundary condition, as described in the published papers (https://doi.org/10.1016/j.coastaleng.2024.104569), (https://doi.org/10.1063/5.0236386) and (https://doi.org/10.1002/nme.5608). Please cite these papers and give a comment on the viscous scheme of SPH.

**Answer:** We appreciate your insightful suggestion regarding the artificial viscous scheme for free surface flows. In this study, the physical viscous scheme (Morris’ formulation [41], Eq. 6) was adopted to prioritize numerical stability and direct alignment with the Navier-Stokes equations, as validated in Section 3.1 (square droplet oscillation) and Section 3.2 (droplet impact on hydrophobic walls). While artificial viscosity is widely used to suppress numerical oscillations near free surfaces, our tests confirmed that the current scheme sufficiently stabilizes simulations without compromising accuracy for the studied cases. Artificial viscosity demonstrates notable advantages in complex free-surface scenarios such as wave breaking and splash [2–4]. We recognize its potential value and will systematically compare both methodologies in subsequent studies to optimize the model and expand its applicability to broader computational domains.

**References:**

[41] Morris J P, Fox P J, Zhu Y. Modeling low Reynolds number incompressible flows using SPH, Journal of Computational Physics 136 (1997) 214-226.

[28] He, F., et al., Multi-phase SPH-FDM and experimental investigations on the hydrodynamics of an oscillating water column wave energy device. Coastal Engineering, 2024. 192: p. 104569. https://doi.org/10.1016/j.coastaleng.2024.104569

[29] Wang, X.J., et al., Study on hydrodynamic characteristics of multiple fish based on smoothed particle hydrodynamics. Physics of Fluids, 2024. 36(11): p. 111915.

https://doi.org/10.1063/5.0236386

[30] Huang, C., Zhang, D. H., Shi, Y. X., Si, Y. L., & Huang, B. (2018). Coupled finite particle method with a modified particle shifting technology. International Journal for Numerical Methods in Engineering, 113(2), 179-207. https://doi.org/10.1002/nme.5608

3. In Eq. (7), what does the variable mean?

**Answer**: Following your suggestions, the variables in Eq. (7) have been clarified and incorporated into the revised manuscript.

$$\frac{d\mathbf{v}_{i}}{dt}=\mathbf{g}_{i}-\sum_{j=1}^{n} m_{j}\left(\frac{P_{i}}{\rho_{i}^{2}}+\frac{P_{j}}{\rho_{j}^{2}}\right)\nabla_{i}W_{ij}\text{ }+\sum_{j} \frac{2m_{j}\mu\mathbf{r}_{ij}\cdot\nabla_{i}W_{ij}}{\rho_{i}\rho_{j}\left(r_{\text{ij}}^{2}+\eta\right)}\mathbf{v}_{ij}\text{ }-\sum_{j=1}^{N} m_{j}\left(\frac{\tilde{p}_{i}}{\tilde{\rho}_{i}^{2}}+\frac{\tilde{p}_{j}}{\tilde{\rho}_{j}^{2}}\right)\nabla_{i}\tilde{W}_{ij}$$

where **v** is the fluid velocity, *p* is the fluid pressure, *μ* is the dynamic viscosity of the fluid, and **g** denotes the body force acting on the fluid, such as gravitational force. **v***_ij_*= **v***_i_*-**v***_j_*, **r***_ij_* =**r***_i_*-**r***_j_*, *r_ij_* = |**r***_ij_*|. The term *η* = 0.01h^2^ was added to prevent the singularity of the viscous term when two particles approach each other infinitely. $\tilde{p}$represents the van der Waals stress. $\tilde{\rho}$represents the van der Waals density.

4. In Eq. (8), what does the variable mean?

**Answer:** We have carefully examined Eq. (8) and provided detailed explanations for the variables in equation (8). These revisions have been incorporated into the manuscript.

where $\tilde{p}$represents the van der Waals stress；$k$ denotes the van der Waals stress；$\tilde{\rho}$represents the van der Waals density.

5. How to solve the symbol m in the term a/m?

**Answer:** The symbol m denotes the mass of a single SPH fluid particle (unit: kg), where *m_i_* =*ρ_i_Vi*, *ρ_i_* represents the density of the particle, and *V_i_* represents the volume of the particle. The calculation method for m has been detailed in the manuscript.

6. It is necessary to cite the reference when introducing the DEM method.

**Answer:** In response to the reviewers' suggestions, we have supplemented relevant references when introducing the digital elevation model (DEM) method.

The Discrete Element Method (DEM) was first proposed by Peter Cundall [34] in 1971 as a numerical method for simulating the movement of solid particles in discontinuous media.

**References:**

[34] Cundall P A (1971). A computer model for simulating progressive large scale movements in blocky rock systems, In Proc. Syrup. Int. Rock Mech, 1: 2-8.

7 How to solve the symbols cn and ct in Eq. (14)?

**Answer:** Thank you for your attention to the damping coefficients *c_n_* and *c_t_* in Equation (14). These two parameters are core components of the particle contact force model in the DEM, directly affecting the accuracy of energy dissipation and dynamic response.

(1) Normal damping coefficient *c_n_*

Based on the Hertz-Mindlin contact theory (Mindlin & Deresiewicz, 1953), the expression of *c_n_* is:

$$c_{n}=-2\xi_{n}\sqrt{k_{n}m_{\text{eff}}}$$

where $m_{\text{eff}}=\frac{m_{i}m_{j}}{m_{i}+m_{j}}$ represents the equivalent mass. *ξ_n_* is the normal damping ratio, calculated through the recovery coefficient e:

$$\xi_{n}=\frac{-\ln e}{\sqrt{\pi^{2}+(\ln e)^{2}}}$$

（2）The tangential damping coefficient *c_t_* is generally proportional to the normal damping. $c_{t}=\eta\cdot c_{n}$，the scaling factor *η*∈[0.5~1.0] ( Cundall & Strack,1979).

We have supplemented the solution process of *c_n_* and *c_t_* in the revised manuscript.

8. what does the variable c in Eq. (15) mean? And, how to solve the symbols k and m in Eq. (15)?

**Answer:** Thank you for your careful review of the formula. Based on the Hertz-Mindlin contact theory (Mindlin & Deresiewicz, 1953), the c in formula (15) is the damping coefficient, where k is the particle stiffness and m is the equivalent mass. We have made necessary modifications to formula 15 to make it clearer.

$$c_{n}=-2\xi_{n}\sqrt{k_{n}m_{\text{eff}}}$$

where $m_{\text{eff}}=\frac{m_{i}m_{j}}{m_{i}+m_{j}}$ represents the equivalent mass. *ξ_n_* is the normal damping ratio, calculated through the recovery coefficient e:

$\xi_{n}=\frac{-\ln e}{\sqrt{\pi^{2}+(\ln e)^{2}}}$ (16)

（2）The tangential damping coefficient *c_t_* is generally proportional to the normal damping.

$c_{t}=\eta\cdot c_{n}$ (17)

the scaling factor *η*∈[0.5~1.0] ( Cundall & Strack,1979).

**References:**

[35] Mindlin, R. D., & Deresiewicz, H. (1953). Elastic spheres in contact under varying oblique forces. Journal of Applied Mechanics.

[36] Cundall, P. A., & Strack, O. D. L. (1979). A discrete numerical model for granular assemblies. Géotechnique.

9. I think the right term of Eq. (19) is equal to μft. Why does the authors use a complex form to express the term of Eq. (19)?

**Answer：**Thank you for your thorough review of Eq. (19). We have carefully examined Eqs. 18 and 19 and identified a typographical error in Eq. 18. This correction has been incorporated into the revised manuscript. The modifications are as follows:

If the two particles are not in contact, then both normal and shear contact forces are set to zero according to the criterion of no normal strength in tension. Otherwise, calculating the maximum allowable shear contact force $\text{ft}_{\text{max}}$ according to Coulomb friction law:

$$\text{ft}_{\text{max}}\text{=}\mu\text{fn}$$

where *µ* is the smaller friction coefficient of the two contact particles.

If, that is, when the magnitude of the tangential contact force exceeds the maximum allowable tangential contact force, the tangential contact force is reduced to the limit value while retaining its sign：

$$\text{ft=ft}\frac{\mu\text{fn}}{\left|\text{ft}\right|}$$

10. For Eqs. (20), (21), (22), (23) and (24), the symbols used to represent solid and fluid particles are too mixed. Please carefully review and modify these formulas.

**Answer:** Thank you for your suggestion. We have modified the notation in equations (20)-(24), where the subscripts for fluid particles are denoted by *i* and *j*, and those for solid particles are denoted by *α* and *β*.

$$\varsigma=\left(\frac{1}{Sa}-0.5\right)\varsigma'$$

$$Sa=\sum_{j=1}^{NL} \frac{m_{j}}{\rho_{j}}W_{ij}$$

11. For figure 1, why is the evolution of the surface by the current method different from the published results? Is it possible that the difference is caused by the viscous discrete format? I suggest that the authors check it carefully.

**Answer:** We appreciate the reviewer’s critical observation regarding the differences in surface evolution between our method and published results in Fig. 1, as well as the suggestion to investigate the role of the viscous term formulation. Below, we provide a detailed analysis and proposed actions to address this concern.

The simulation in Fig. 1 demonstrates that the proposed SPH method achieves higher symmetry during the free deformation of a square droplet under surface tension compared to other methods. However, transient discrepancies in shape evolution (e.g., intermediate deformation stages) may arise from the following factors:

**a. Viscous Term Formulation:**

The viscous force in the SPH momentum equation (Eq. 6) follows the formulation by Morris [41]. This differs from some published methods (e.g.,artificial viscosity or sub-particle scale models), which may introduce additional damping or smoothing.

**b. Surface Tension Implementation:**

The proposed model incorporates surface tension via a Van der Waals stress term (Eq. 7–9), avoiding direct interparticle force calculations. In contrast, classical approaches like the Continuum Surface Force (CSF) model or Inter-Particle Force (IIF) model explicitly compute curvature-dependent forces, which may lead to variations in transient shape dynamics.

**c. Kernel Function and Smoothing Length:**

The cubic spline kernel (*h*=1.3Δ*x*) and spatial resolution (Δ*x*=0.04mm) influence particle interactions. Coarser resolutions or alternative kernels (e.g., Wendland) may alter the balance between surface tension and viscous forces during deformation.

We thank the reviewer for highlighting this issue, and we will introduce the delta-SPH model and optimize the viscosity parameters to compare the influence of different viscous discrete schemes on the results.

Thanks to you for your comments and suggestions again.

The authors tried our best to improve the manuscript and made some changes in the manuscript. These changes will not influence the content and framework of the paper. And here we did not list the changes but marked in yellow highlight in revised manuscript.

The authors appreciate for Editors and Reviewers’ warm work earnestly, and hope that the correction will meet with approval.
